# Cucurbitacin B Induces the Lysosomal Degradation of EGFR and Suppresses the CIP2A/PP2A/Akt Signaling Axis in Gefitinib-Resistant Non-Small Cell Lung Cancer

**DOI:** 10.3390/molecules24030647

**Published:** 2019-02-12

**Authors:** Pengfei Liu, Yuchen Xiang, Xuewen Liu, Te Zhang, Rui Yang, Sen Chen, Li Xu, Qingqing Yu, Huzi Zhao, Liang Zhang, Ying Liu, Yuan Si

**Affiliations:** 1 Laboratory of Molecular Target Therapy of Cancer, Institute of Basic Medical Sciences, Hubei University of Medicine, Shiyan 442000, China; LiuPF_001@163.com (P.L.); xiangyc4026@163.com (Y.X.); liuxw1110@163.com (X.L.); yangr0512@163.com (R.Y.); sener_chen@163.com (S.C.); zhaohz07@163.com (H.Z.); zl_19820321@163.com (L.Z.); 2 Laboratory of Molecular Target Therapy of Cancer, Biomedical Research Institute, Hubei University of Medicine, Shiyan 442000, China; zhangte519@163.com (T.Z.); xuli103028@sina.com (L.X.); 18064081841@163.com (Q.Y.); 3 Hubei Key Laboratory of Wudang Local Chinese Medicine Research and Institute of Medicinal Chemistry, Hubei University of Medicine, Shiyan 442000, China

**Keywords:** Cucurbitacin B, gefitinib-resistant NSCLC, EGFR, lysosomal degradation, CIP2A

## Abstract

Non-small cell lung cancer (NSCLC) patients carrying an epidermal growth factor receptor (EGFR) mutation are initially sensitive to EGFR-tyrosine kinase inhibitors (TKIs) treatment, but soon develop an acquired resistance. The treatment effect of EGFR-TKIs-resistant NSCLC patients still faces challenges. Cucurbitacin B (CuB), a triterpene hydrocarbon compound isolated from plants of various families and genera, elicits anticancer effects in a variety of cancer types. However, whether CuB is a viable treatment option for gefitinib-resistant (GR) NSCLC remains unclear. Here, we investigated the anticancer effects and underlying mechanisms of CuB. We report that CuB inhibited the growth and invasion of GR NSCLC cells and induced apoptosis. The inhibitory effect of CuB occurred through its promotion of the lysosomal degradation of EGFR and the downregulation of the cancerous inhibitor of protein phosphatase 2A/protein phosphatase 2A/Akt (CIP2A/PP2A/Akt) signaling axis. CuB and cisplatin synergistically inhibited tumor growth. A xenograft tumor model indicated that CuB inhibited tumor growth in vivo. Immunohistochemistry results further demonstrated that CuB decreased EGFR and CIP2A levels in vivo. These findings suggested that CuB could suppress the growth and invasion of GR NSCLC cells by inducing the lysosomal degradation of EGFR and by downregulating the CIP2A/PP2A/Akt signaling axis. Thus, CuB may be a new drug candidate for the treatment of GR NSCLC.

## 1. Introduction

Lung cancer is the most commonly diagnosed cancer and the leading cause of cancer-related death. An estimated two million new lung cancer cases were recorded in 2018, and these cases account for approximately 11.6% of the total number of cancer cases [1]. Non-small cell lung cancer (NSCLC) accounts for the majority (80%) of lung cancer cases [2]. Although most NSCLC patients initially respond to chemotherapy, they gradually become drug-resistant, which in turn leads to cancer recurrence and poor prognosis [3]. Gefitinib and erlotinib are epidermal growth factor receptor-tyrosine kinase inhibitors (EGFR-TKIs). The treatment effect of EGFR-TKIs is significant for NSCLC patients with EGFR activating mutations (such as exon 19 deletion and the L858R point mutation). However, cancer cells often develop TKI resistance, which in turn causes tumor recurrence [4]. Therefore, acquired EGFR-TKI resistance is a clinical problem that needs to be solved. Patients with acquired resistance to gefitinib or erlotinib have acquired a second mutation in exon 20 of the EGFR gene, resulting in the replacement of threonine at position 790 in the protein kinase domain with methionine (T790M). Threonine 790 is an important amino acid residue in EGFR that occupies the adenosine triphosphate (ATP)-binding pocket adjacent to the ATP-binding cleft, and it determines the binding specificity of the inhibitor. The replacement of Thr790 by Met increases the affinity for ATP and reduces the binding of any ATP-competitive kinase inhibitors. [5]. Thus, treatment strategies for secondary mutations of EGFR (T790M) should be developed to overcome EGFR-TKI resistance, which would benefit NSCLC patients.

In the past 10 years, the cancerous inhibitor of protein phosphatase 2A (CIP2A) has been increasingly recognized as a key oncoprotein in several human malignancies, including myeloma [6], breast cancer [7], gastric cancer [8], glioma [9], and colorectal cancer [10]. Previous independent studies have shown that abnormal CIP2A overexpression is associated with tumor growth, anti-apoptotic effects, drug resistance, metastasis, and poor prognosis of the malignant tumors mentioned above. Additionally, CIP2A is involved in the occurrence of NSCLC, and the overexpression of CIP2A is associated with cigarette smoking and poor prognosis [11,12]. CIP2A is an endogenous inhibitor of the key tumor suppressor protein phosphatase 2A (PP2A) [13]. A previous review proposed an interactive regulatory network (carcinogenic nexus) involving CIP2A [14]. In this network, CIP2A interacts with various key cellular protein/transcription factors or components of key oncogenic signaling pathways through direct interaction or through indirect CIP2A-PP2A interactions. The primary role of CIP2A in the “carcinogenic nexus” is the inhibition of another important associated component, PP2A. PP2A is a tumor suppressor that regulates homeostasis by inhibiting intracellular signaling pathways that are driven by the constitutive activation of multiple kinases [15]. Mutations leading to the abnormal expression of PP2A scaffolds and regulatory subunits are common in many human cancers [16]. Therefore, based on its tumor suppressive properties, the reactivation of PP2A is a potential strategy for cancer treatment [17,18]. Targeting the oncoprotein CIP2A is an important strategy to reactivate PP2A to treat cancer.

Cucurbitacin is a natural tetracyclic triterpenoid compound mainly found in *Cucurbitaceae* [9]. In China and India, the use of *Cucurbitaceae* as an herbal medicine is based on its different biological activities, such as its anti-diabetic, anti-inflammatory, and anti-cancerous activities against different cancer types [19,20]. Cucurbitacin B (CuB), one of the most important members of the cucurbitacin family, has been shown to have antiplasmodial, immunomodulatory, hepatoprotective, antioxidant, cardiovascular, anthelmintic, anti-inflammatory, and anti-fertility activities [21]. Recently, several studies have reported that CuB-mediated anti-cancer activities are mainly mediated through the activation of apoptosis, cell cycle arrest, and autophagy, as well as through the suppression of the STAT3 and Raf/MEK/ERK pathways [22]. However, no study has examined the efficacy of CuB in gefitinib-resistant (GR) NSCLC. This study is the first to report that CuB induces EGFR degradation and has CIP2A/PP2A/Akt inhibitory activities in GR NSCLC cells. 

## 2. Materials and Methods

### 2.1. Reagents

Cucurbitacin B (CuB) with a purity of up to 98% was purchased from Shanghai Yuanye Bio-Technology Co., Ltd. (Shanghai, China). CuB was dissolved in DMSO, (Sigma-Aldrich; Merck Millipore, Darmstadt, Germany) at a stock solution of 40 mM and stored at –20 °C. 

### 2.2. Cell Culture

Human gefitinib-resistant NSCLC cell lines A549, NCI-H1299 (H1299), NCI-H1975 (H1975), and NCI-H820 (H820), and human normal lung epithelial cell line (16-HBE) were obtained from American Type Culture Collection (ATCC, Manassas, VA, USA). A549 and H1299 harbor wild-type EGFR. H1975 harbors L858R and T790M double mutation on EGFR, and H820 harbors exon 19 in frame deletion and T790M double mutation on EGFR. A549, H1299, and 16-HBE cells were cultured in Dulbecco modified Eagle medium (DMEM, Gibco; Thermo Fisher Scientific, Inc., Waltham, MA, USA). H1975 and H820 cells were cultured in Roswell Park Memorial Institute (RPMI) 1640 medium (Gibco; Thermo Fisher Scientific, Inc.). DMEM and RPMI 1640 medium were supplemented with 10% fetal bovine serum (FBS; HyClone, Logan, UT, USA), 100 U/mL penicillin, and 100 μg/mL streptomycin (both from Gibco; Thermo Fisher Scientific, Inc.), and cultured in a humidified atmosphere with 5% CO_2_ at 37 °C.

### 2.3. Cytotoxic Assay and Cell Viability

Cells were seeded into a 96-well plate and pre-cultured for 24 h, and then treated with CuB or geftinib for 24 h. Cell cytotoxicity was determined by an 3-(4,5-dimethylthiazol-2-yl)-2,5-diphenyltetrazolium bromide (MTT) assay. The absorbance was measured at 570 nm by an automated microplated reader (BioTek Instruments, Inc., Winooski, VT, USA), and the cell death rate was calculated as follows: inhibition rate (%) = (average A_570_ of the control group − average A_570_ of the experimental group)/(average A_570_ of the control group − average A_570_ of the blank group) × 100%. Cell viability was estimated by trypan blue dye exclusion.

### 2.4. Soft-Agar Colony Formation Assay

Cells were suspended in 1 ml of RPMI 1640 containing 0.3% low-melting-point agarose (Amresco, Cleveland, OH, USA) and 10% FBS, and plated on a bottom layer containing 0.6% agarose and 10% FBS in a six-well plate in triplicate. After two weeks, plates were stained with 0.2% gentian violet and the colonies were counted under a light microscope (IX70; Olympus Corporation, Tokyo, Japan) after two weeks.

### 2.5. Invasion Assay

An invasion assay was carried out using a 24-well plate (Corning, Inc., Corning, NY, USA). A polyvinyl-pyrrolidone-free polycarbonate filter (8 μm pore size) (Corning) was coated with matrigel (BD Biosciences, Franklin Lakes, NJ, USA). The lower chamber was filled with medium containing 20% FBS as a chemoattractant. The coated filter and upper chamber were laid over the lower chamber. Cells (1 × 10^4^ cells/well) were seeded onto the upper chamber wells. After incubation for 20 h at 37 °C, the filter was fixed and stained with 2% ethanol containing 0.2% crystal violet (15 min). After being dried, the stained cells were enumerated under a light microscope at 10× objective. For quantification, the invaded stained cells on the other side of the membrane were extracted with 33% acetic acid. The absorbance of the eluted stain was determined at 570 nm.

### 2.6. Wound Healing Assay

Cells (4 × 10^5^ cells/2 mL) were seeded in a six-well plate and incubated at 37 °C until 90% to 100% confluence. After this, the confluent cells were scratched with a 200 μL pipet tip, followed by washing with PBS, and then treated with serum free medium. After 24 h of incubation, the cells were fixed and stained with 2% ethanol containing 0.2% crystal violet powder (15 min), and randomly chosen fields were photographed under a light microscope at 4× objective. The number of cells that had migrated into the scratched area was calculated.

### 2.7. Western Blot

Cell pellets were lysed in radioimmunoprecipitation assay (RIPA) buffer containing 50 mM Tris at pH 8.0, 150 mM NaCl, 0.1% sodium lauryl sulfate (SDS), 0.5% deoxycholate, 1% nonidet P-40 (NP-40), 1 mM DL-dithiothreitol (DTT), 1 mM NaF, 1 mMNaVO_3_, 1 mM phenylmethanesulfonyl fluoride (PMSF, Sigma-Aldrich; Merck Millipore, Darmstadt, Germany), and 1% protease inhibitors cocktail (Merck, Millipore). Lysates were normalized for total protein (25 µg) and loaded on 8% to 12% sodium dodecyl sulfate polyacrylamide gel, electrophoresed, and transferred to a PVDF membrane (Millipore, Kenilworth, NJ, USA), followed by blocking with 5% skimmed milk at room temperature for 1 h. The membrane was incubated with primary antibodies overnight at 4 °C and rinsed with Tris-buffered saline with Tween 20. The primary antibodies used were anti-caspase-3 (1:1000 dilution; catalog no. 9662), anti-caspase-8 (1:1000 dilution; catalog no. 9746), anti-poly(adenosine diphosphate (ADP) ribose) polymerase (PARP; 1:1000 dilution; catalog no. 9542), anti-EGFR (1:1000 dilution; catalog no. 4267), anti-ERK1/2 (1:1000 dilution; catalog no. 9102), anti-phospho-ERK1/2 (Thr202/Tyr204) (1:1000 dilution; catalog no. 9101), anti-PP2A (1:1000 dilution; catalog no. 2038) (all Cell Signaling Technology, Inc., Danvers, MA, USA), anti-CIP2A (1:500 dilution; catalog no. sc-80662), anti-phospho-Akt (S473) (1:500 dilution; catalog no. sc-7985), anti-Akt (1:500 dilution; catalog no. sc-8312) (all Santa Cruz Biotechnology, Inc., Dallas, TX, USA), and anti-glyceraldehyde-3-phosphate dehydrogenase (GAPDH, 1:5000 dilution; catalog no. M20006; Abmart, Shanghai, China). The blots were then washed and incubated with horseradish peroxidase (HRP)-conjugated secondary antibody (1:10,000 dilution; catalog no. E030120-01 and E030110-01; EarthOx, LLC, San Francisco, CA, USA) for 1.5 h at room temperature. Detection was performed by using a SuperSignal^®^ West Pico Trial kit (catalog no. QA210131; Pierce Biotechnology, Inc., Rockford, IL, USA) [23]. The defined sections of the film were scanned for image capture and quantification using Adobe Photoshop software (CS4, Adobe Systems Incorporated, California, USA) and Image J software (National Institutes of Health, Bethesda, MD, USA).

### 2.8. Quantitative Polymerase Chain Reaction

The expression level of the *EGFR* gene was examined by quantitative polymerase chain reaction (QPCR). GAPDH was used as an endogenous control for each sample. Total RNA from SW620 or HT29 cells or patients’ tissues was extracted using TRIzol reagent (Invitrogen; Thermo Fisher Scientifc, Inc.,) according to the manufacturer’s protocols. Total RNA (2 µg) and the ReverTra Ace qPCR real time kit (Toyobo Life Science, Osaka, Japan) were used for the QPCR analysis of *CIP2A*. Reverse transcription occurred at 37 °C for 15 min and 98 °C for 5 min, with storage at –20 °C. RNA (2 µg), 4 μL 5 RT Buffer, 1 μL RT Enzyme mix, 1 μL Primer mix, and Nuclease-free Water were mixed to a 20 μL total volume. The primers used in this study were as follows: *EGFR* forward primer: 5′- TTGTTCCTCACTGCTGTTCAC-3′ and *EGFR* reverse primer: 5′-GTCCATCATCTGTCTCCTTTC-3′; and *GAPDH* forward, 5′-TGTTGCCATCAATGACCCCTT-3′ and reverse, 5′-CTCCACGACGTACTCAGCG-3′. QPCR was performed using an ABI StepOnePlus™ Real-Time PCR system (Applied Biosystems; Thermo Fisher Scientific, Inc.) with the Power SYBR^®^ Green PCR Master mix (Toyobo Life Science). SYBR Green PCR Master Mix (10 μL), forward and reverse primers (200 nM), a cDNA template (100 ng), and doubly-distilled H_2_O were mixed to a 20 μL total volume. PCR conditions consisted of the following: 95 °C for 3 min, 95 °C for 15 s, and 60 °C for 1 min, for 40 cycles. The threshold cycle for each sample was selected from the linear range and converted to a starting quantity by interpolation from a standard curve generated on the same plate for each set of primers. The *CIP2A* mRNA levels were evaluated using the 2^−ΔΔCq^ method, standardized to levels of GAPDH amplification [24]. Each test was performed in triplicate.

### 2.9. Immunofluorescence Staining

H1975 cells were incubated in the presence or absence of CuB for 24 h. Cells were then fixed and penetrated. Primary antibodies were added at a dilution of 1:50 and incubated with cells at 4 °C overnight. Dylight 488 or Dylight 594-conjugated secondary antibodies (EarthOx, LLC, San Francisco, CA, USA) were diluted 1:500 in 3% BSA in PBS for 1.5 h at room temperature. For visualization of the cell nucleus, 4′,6-diamidino-2-phenylindole (DAPI) was used. Sections were observed using an Olympus laser scanning confocal microscope with imaging software (Olympus Fluoview FV-1000, Tokyo, Japan).

### 2.10. PP2A Activity Assay

PP2A phosphatase activity was tested using a PP2A immunoprecipitation phosphatase assay kit (Upstate Biotechnology, Inc., Lake Placid, NY, USA). According to the manufacturer’s instructions, 100 μg protein isolated from the cells and 4 μg anti-PP2A monoclonal antibody (1:100 dilution; catalog no. 2038; Cell Signaling Technology, Inc.) were incubated together at 4 °C overnight. Protein A agarose beads (40 μL) were added to the mixture and incubated at 4 °C for 2 h, and the beads were then collected and washed three times with 700 μL ice-cold TBS and once with 500 μL Ser/Thr Assay Buffer (Upstate Biotechnology, Inc.). The beads were further incubated with 750 mM phosphopeptide in assay buffer at 30 °C for 10 min with continuous agitation. Malachite Green Phosphate Detection Solution (100 μL) was added and the absorbance at 650 nm was measured, as described previously [25].

### 2.11. Transfection of DNA

The pOTENT-1-CIP2A expression plasmid was purchased from Youbio Co. (Changcha, China). The pOTENT-1-CIP2A plasmid (1 μg/μL) was transfected into GR NSCLC cells using Lipofectamine® 3000 transfection reagent (Invitrogen; Thermo Fisher Scientific, Inc.) following the manufacturer’s protocols.

### 2.12. Drug Combination Assay

Drug combination is widely used in cancer treatment to achieve a synergistic therapeutic effect and overcome drug resistance in clinics. To estimate the effect of CuB and DDP combination, the combination index (CI) was calculated by the Chou-Talalay equation [23]. H1975 or H820 cells were seeded in 96-well plates. Drugs were added alone or together at an indicated concentration. The inhibition effect was measured by an MTT assay, as mentioned above. The formula of CI = (D)CuB/(Dx)CuB + (D)DDP/(Dx)DDP. (D: the doses of compounds CuB or DDP, respectively, necessary to produce the same effect in combination. Dx: the dose of one compound alone required producing an effect). With this formula and the assistance of CalcuSyn software (Version 2.1, Biosoft, Cambridge, UK), the combined effects of the two compounds could be assessed as follows: CI < 1 indicates synergism; CI = 1 indicates additive effect; and CI > 1 indicates Antagonism.

### 2.13. Human NSCLC Xenograft Experiments

Equal numbers of female and male (*n* = 24), five-week-old, nude immunodeficient mice (nu/nu) (weighing ~16 g) were purchased from Hunan SJA Laboratory Animal Co., Ltd. (Changsha, China), and maintained and monitored in a specific pathogen-free environment (temperature 22~24 °C, barrier environment, 12 h/12 h, sterile water, full nutritive feed). All animal studies were conducted according to protocols approved by the Hubei University of Medicine Animal Care and Use Committee, complying with the rules of Regulations for the Administration of Affairs Concerning Experimental Animals (Approved by the State Council of China, No. SYXK (Hubei) 2016-0031). The mice were injected subcutaneously with GR NSCLC H1975 cells (2.5 × 10^6^) suspended in 100 μL RPMI 1640 medium into the right flank of each mouse. Treatments were started when the tumors reached a palpable size. Caliper measurements of the longest perpendicular tumor diameters were performed twice a week to estimate the tumor volume, using the following formula: 4π/3 × (width/2)^2^ × (length/2), representing the three-dimensional volume of an ellipse. Animals were sacrificed when tumors reached 1.5 cm or if the mice appeared moribund to prevent unnecessary morbidity to the mice. At the time of the animals’ death, tumors were excised for immunohistochemistry.

### 2.14. Immunohistochemistry of Tissues

Formalin-fixed, paraffin-embedded tissues from mice were selected for immunohistochemical examination by using an indirect immunoperoxidase method. The antibodies used for immunohistochemical staining were EGFR and CIP2A.

### 2.15. Statistical Analysis

All statistical analyses were conducted using GraphPad Prism 5 (GraphPad Software, Inc., La Jolla, CA, USA) and SPSS 22.0 software for Windows (IBM Corp., Armonk, NY, USA). Results from three independent experiments were presented as the mean ± standard deviation, unless otherwise noted. Statistically significant values were compared using Student’s t-test of unpaired data or one-way analysis of variance and Bonferroni’s post hoc test, *P* < 0.05 was used to indicate a statistically significant difference.

## 3. Results

### 3.1. CuB Induces Cytotoxicity in Gefitinib-Resistant Non-Small Cell Lung Cancer Cells

**Figure 1:**
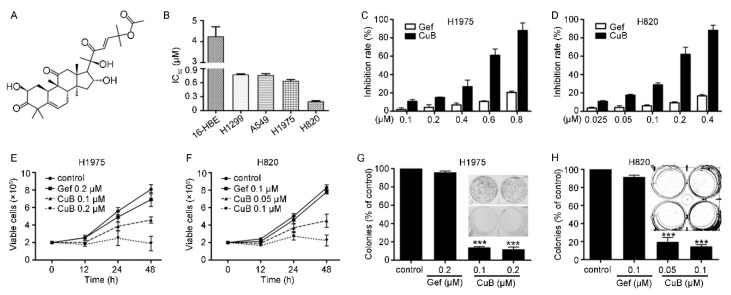
Cucurbitacin (CuB) inhibits gefitinib-resistant non-small cell lung cancer cells (GR-NSCLC) cells. (**A**): Chemical structure of CuB. (**B**): The IC_50_ of CuB for indicated cell lines. (**C**–**D**): H1975 and H820 cells were treated with increasing concentration of CuB or gefitinib for 24 h, and analyzed by MTT assay. Gef: gefitinib. (**E**–**F**): Inhibitory effects of CuB on cell viability of H1975 and H820 cells assayed by trypan blue exclusion assay. (**G**–**H**): The colony formation assays of H1975 and H820 cells treated with CuB at indicated concentration.

The effect of CuB on cell proliferation was determined using four GR NSCLC cell lines, namely, H1975, H820, A549, and H1299, and one normal lung epithelial cell line, 16-HBE. These four GR NSCLC cell lines have different EGFR gene mutations. The H1975 cell line has a double mutation of L858R and T790M in EGFR, and the H820 cell line has a frameshift deletion of exon 19 and a T790M mutation in EGFR. Both the A549 and H1299 cell lines express the wild-type EGFR protein. MTT assays suggested that CuB was moderately cytotoxic to all four cell lines, with an IC_50_ value between 4.23 μM and 0.19 μM (Table 1). As shown in Figure 1B–D, CuB was effective in suppressing the proliferation of GR NSCLC (H1975 and H820) cells. Interestingly, CuB had the weakest inhibitory effect on normal lung epithelial cells (16-HBE). Trypan blue exclusion assays suggested that CuB decreased the viability of H1975 (Figure 1E) and H820 (Figure 1F) cells in a dose- and time-dependent manner. We next determined the effect of CuB on cell colony formation activity, and we found that CuB markedly inhibited the clonogenic ability of H1975 (Figure 1G) and H820 (Figure 1H) cells. These data indicated that CuB suppressed the anchorage-dependent (growth) and anchorage-independent (clonogenic ability) proliferation of GR NSCLC cells. In the remainder of the study, the CuB dose that was selected for inhibition was less than 30% to ensure cellular integrity.

### 3.2. CuB Inhibits Invasion and Migration and Induces Caspase-Dependent Apoptosis of Gefitinib-Resistant Non-Small Cell Lung Cancer Cells

**Figure 2:**
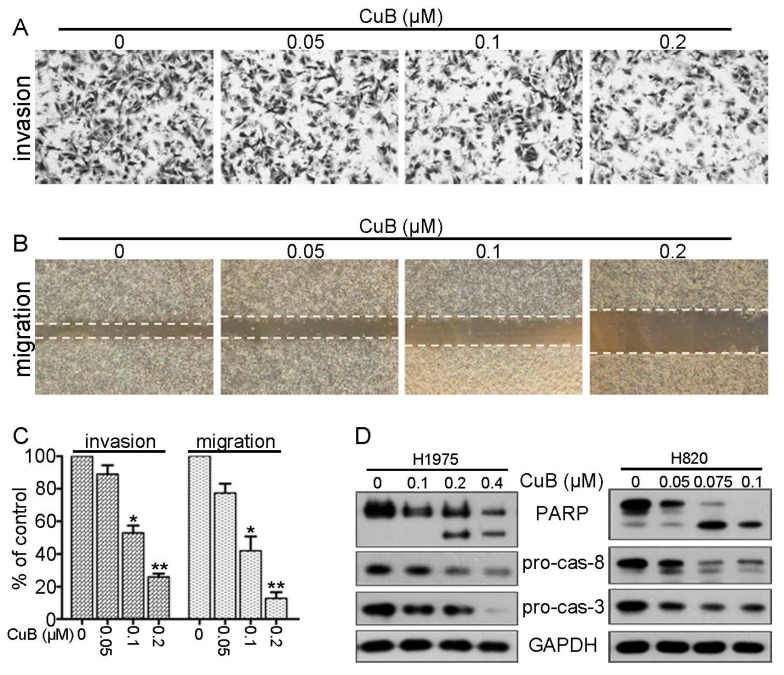
CuB reduces invasive behavior and induces apoptosis of GR NSCLC cells. (**A**) Invasion assay was carried out using modified 24-well microchemotaxis chambers. H1975 cells were pretreated with CuB for 30 min. (**B**) Confluent H1975 cells were scratched and then treated with CuB in a basic medium for 24 h. (**C**) Statistical results of Figure 2A,B. Data are shown as the mean ± SD of three independent experiments.* *P* < 0.05; ** *P* < 0.01 vs. 0 μM. (**D**) H1975 and H820 cells were treated with increasing concentrations of CuB for 24 h. Western blot was performed using antibodies indicated. GAPDH was used as the loading control.

We investigated whether CuB suppressed the invasive behavior of H1975 cells. An invasion assay suggested that low doses of CuB (0–0.1 µM) inhibited the invasion of H1975 cells (Figure 2A,C). Furthermore, the wound healing assay suggested that CuB markedly decreased H1975 cell migration in a dosage-dependent manner (Figure 2B,C). These data indicated that CuB inhibited the invasive behavior of GR NSCLC cells at relatively lowly cytotoxic concentrations.

We next determined the effect of CuB on apoptosis in GR NSCLC cells. Western blot analysis suggested that CuB induced a marked increase in the active form of both caspase-8 (casp-8) and caspase-3 (casp-3) and induced the cleavage of poly(ADP-ribose) polymerase (PARP) in H1975 cells and H820 cells in a dose-dependent manner (Figure 2D). These data suggested that CuB induced caspase-dependent apoptosis in GR NSCLC cells.

### 3.3. CuB Induces the Lysosomal Degradation of EGFR and, thus, Inhibits ERK Signaling

**Figure 3:**
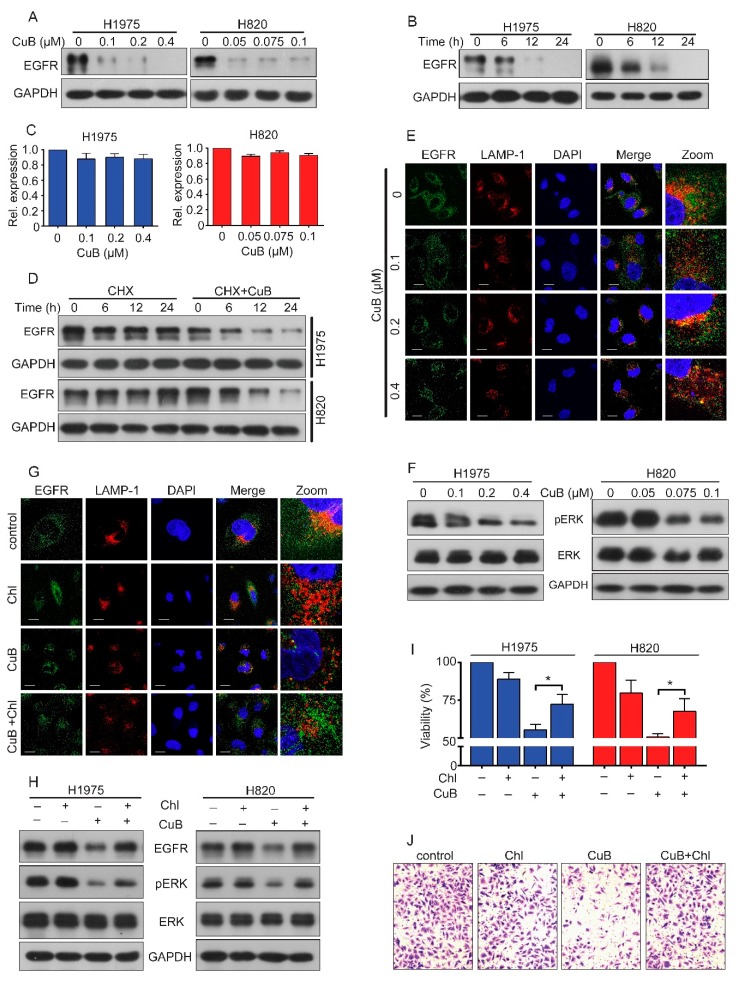
CuB induces lysosomal degradation of EGFR and thus inhibits ERK signaling in GR NSCLC cells. (**A**) H1975 and H820 cells were treated with increasing concentrations of CuB for 24 h. Western blot was performed using antibodies indicated. (**B**) H1975 (or H820) cells were treated with 0.2 µM (or 0.075 µM) CuB for the indicated times, and cell lysates were subjected to western blot assay. (**C**) The mRNA level of *EGFR* in H1975 and H820 cells treated with CuB for 24 h was analyzed by QPCR. (**D**) H1975 (or H820) cells were treated with 40 µg/ml cycloheximide (CHX) in the absence or presence of 0.2 µM (or 0.075 µM) CuB for the indicated times, and cell lysates were harvested for western blot assay. (**E**) H1975 cells were exposed to increasing concentrations of CuB for 24 h. For immunofluorescence analysis, cells was stained with an anti-EGFR, anti-LAMP-1 antibodies, and DAPI and observed by confocal microscopy. Scale bar = 20 μm. (**F**) H1975 and H820 cells were treated with increasing concentrations of CuB for 24 h. Western blot was performed using antibodies indicated. (**G**) H1975 cells were pretreated with chloroquine (Chl; 10 μM) for 2 h, followed by addition of CuB (0.4 μM) for 22 h. For immunofluorescence analysis, cells was stained with an anti-EGFR, anti-LAMP-1 antibodies, and DAPI and observed by confocal microscopy. Scale bar = 20 μm. (**H**) H1975 (or H820) cells were pretreated with Chl (10 μM) for 2 h, followed by addition of 0.2 µM (or 0.075 µM) CuB for 22 h. Western blot was performed using antibodies indicated. (**I**) H1975 (or H820) cells were pretreated with Chl (10 μM) for 2 h, followed by addition of 0.5 µM (or 0.15 µM) CuB for 22 h and then analyzed by MTT assay. (**J**) H1975 cells were pretreated with Chl (10 μM) for 2 h, followed by addition of 0.2 µM CuB for 2 h. Invasion assay was carried out using modified 24-well microchemotaxis chambers. * *P* < 0.05.

Since mutated EGFR plays a critical role in the growth and invasion of NSCLC cells, we next determined the effect of CuB on EGFR expression in H1975 and H820 cells. Interestingly, we found that treatment with CuB at 0.1 μM in H1975 cells and 0.05 μM in H820 cells caused the downregulation of EGFR expression at the protein level (Figure 3A). We further showed that CuB caused the downregulation of EGFR in a time-dependent manner (Figure 3B). We next determined whether CuB affected *EGFR* gene transcription by QPCR. The results suggested that CuB had no significant effect on *EGFR* mRNA expression (Figure 3C). These data indicated that CuB may affect EGFR protein stability. Next, we blocked protein synthesis by the protein synthesis inhibitor cycloheximide (CHX) and found that EGFR remained stable after more than 12 h of CHX treatment. However, it was downregulated at 6 h in cells treated with CHX plus CuB (Figure 3D). These data indicated that CuB induced EGFR proteolysis. Previous work has reported that EGFR degradation is mediated by the lysosomal pathway [26]. Immunofluorescence analysis showed an increased colocalization of EGFR and the lysosomal marker lysosomal-associated membrane protein 1 (LAMP-1) (Figure 3E) in CuB-treated H1975 cells, suggesting that CuB promoted EGFR trafficking to lysosomes. Extracellular regulated protein kinases (ERK) are proteins in major downstream signaling of EGFR that promote cell proliferation. Activated ERK translocates to the nucleus and transactivates transcription factors, altering gene expression to promote cell cycle progression and invasion [27,28]. We measured ERK activity in CuB-treated GR NSCLC cells and found that CuB can decrease phosphorylated ERK (pERK) in a dose-dependent manner, without causing clear changes in the total ERK expression in H1975 and H820 cells (Figure 3F). In the presence of the lysosome inhibitor chloroquine (Chl), EGFR accumulation (Figure 3H) and colocalization of EGFR with lysosomes was reduced (Figure 3G), suggesting that CuB promoted the lysosomal degradation of EGFR. In addition, Chl antagonized the inhibitory effect of CuB on cell proliferation (Figure 3I). Furthermore, we compared pERK in cells treated with CuB in the presence and absence of Chl. The data indicated that Chl partially reversed the inhibitory effect of CuB on ERK phosphorylation (Figure 3H) and cell invasion (Figure 3J). These results further suggested that CuB reduced invasion and pERK levels via EGFR degradation.

### 3.4. CuB Downregulates the CIP2A/PP2A/Akt Signaling Axis in Gefitinib-Resistant Non-Small Cell Lung Cancer Cells

**Figure 4:**
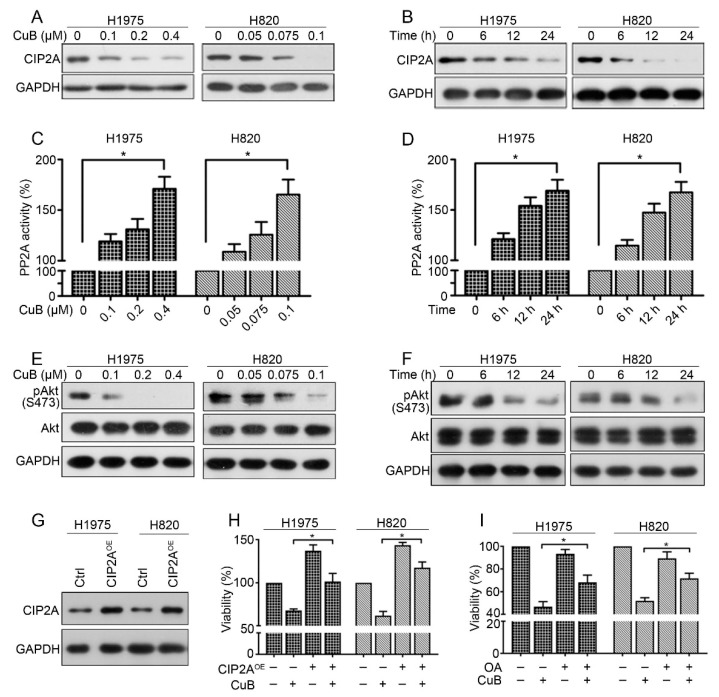
CuB down-regulates CIP2A/PP2A/Akt signal axis in GR NSCLC cells. (**A**) H1975 and H820 cells were treated with increasing concentrations of CuB for 24 h. Western blot was performed using antibodies indicated. (**B**) H1975 (or H820) cells were treated with 0.2 µM (or 0.075 µM) CuB for the indicated times, and cell lysates were subjected to western blot assay. (**C**) H1975 and H820 cells were treated with increasing concentrations of CuB for 24 h. Cell lysates were prepared for detecting PP2A activity, as mentioned before. (**D**) H1975 (or H820) cells were treated with 0.2 µM (or 0.075 µM) CuB for the indicated times, and cell lysates were prepared for detecting PP2A activity, as mentioned before. (**E**) H1975 and H820 cells were treated with increasing concentrations of CuB for 24 h. Western blot was performed using antibodies indicated. (**F**): H1975 (or H820) cells were treated with 0.2 µM (or 0.075 µM) CuB for the indicated times, and cell lysates were subjected to western blot assay. (**G**) H1975 (or H820) cells were transfected with a CIP2A expression plasmid (CIP2A^OE^), and total protein was isolated and then subjected to western blot analysis. (**H**) H1975 (or H820) cells were transfected with the CIP2A^OE^, and then treated with CuB (H1975: 0.4 µM; H820: 0.1 µM), MTT assay was used to detect growth 48 h after transfection. (**I**) H1975 (or H820) cells were treated with CuB (H1975: 0.45 µM; H820: 0.15 µM) and/or OA (50 nM) for 24 h and analyzed by MTT assay. * *P* < 0.05.

In our previous work, we reported that CIP2A plays an important role in the proliferation and aggressiveness of NSCLC and the natural compound oridonin could downregulate CIP2A levels in GR NSCLC cells [20]. Here, we reported that treatment with CuB at 0.1–0.4 μM for 24 h could downregulate CIP2A expression in H1975 cells (0.05–0.1 µM in H820 cells) (Figure 4A). As shown in the left panel, CIP2A protein expression was decreased in H1975 cells exposed to 0.2 μM of CuB. Furthermore, treatment of H820 cells with 0.075 μM of CuB caused an apparent downregulation of CIP2A. We also demonstrated that CuB induced the downregulation of CIP2A in a time-dependent manner (Figure 4B). CIP2A is an endogenous inhibitor of the tumor suppressor protein phosphatase 2A (PP2A) and is highly expressed in a variety of tumors [29]. Next, we examined the activity of PP2A and found that PP2A activity was significantly increased in H1975 and H820 cells after CuB treatment (Figure 4C,D). The expression and activation of Akt downstream of PP2A was further examined, and we found that CuB downregulated Akt phosphorylation (pAkt) in H1975 and H820 cells (Figure 4E,F), and the total Akt level did not clearly change. These results suggest that CuB downregulated the CIP2A/PP2A/Akt pathway in GR NSCLC cells.

To further confirm the role of the CIP2A pathway in mediating the growth inhibition of GR NSCLC cells by CuB, we generated H1975 and H820 cells that overexpressed a CIP2A (CIP2A^OE^) plasmid by transient transfection (Figure 4G). Compared with that of wild-type cells, the proliferation of CIP2A^OE^ cells significantly increased (Figure 4H). Notably, CIP2A overexpression significantly antagonized CuB-induced growth inhibition (Figure 4H). These findings demonstrated that CIP2A may play a critical role in CuB-triggered GR NSCLC growth. We subsequently examined whether PP2A inhibition would alter cellular sensitivity to CuB. Okadaic acid (OA), a PP2A inhibitor, was applied to H1975 and H820 cells with or without CuB treatment. Pretreatment with OA antagonized the effects of CuB on growth in H1975 and H820 cells (Figure 4I). Thus, we confirmed that CuB induced cell growth inhibition, at least in part, by downregulating the CIP2A/PP2A/Akt pathway.

**Figure 5:**
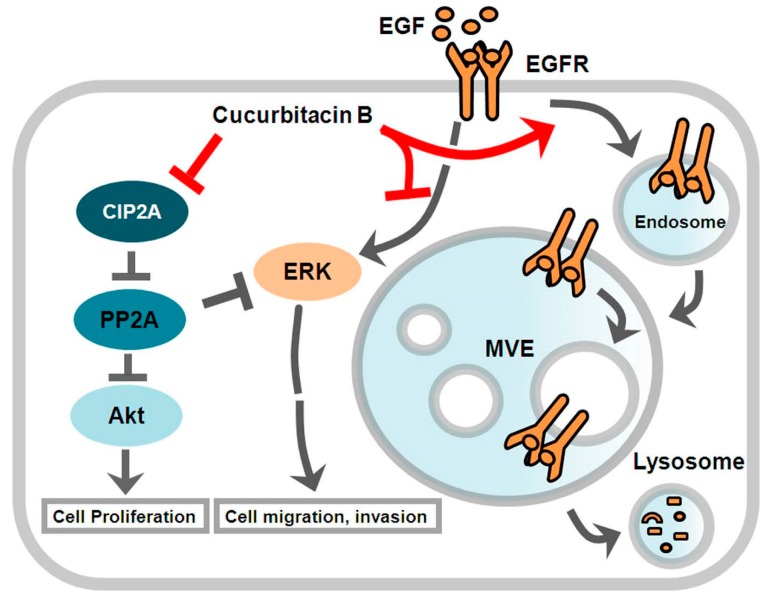
Diagram of CuB blockage possible mechanism in GR NSCLC cells.

Based on the results presented above, we concluded that CuB inhibited GR NSCLC growth and induced apoptosis by inducing the lysosomal degradation of EGFR and by downregulating the CIP2A/PP2A/Akt signaling axis (Figure 5).

### 3.5. CuB and Cisplatin Synergistically Inhibit the Proliferation and Apoptosis of Gefitinib-Resistant Non-Small Cell Lung Cancer Cells

**Figure 6:**
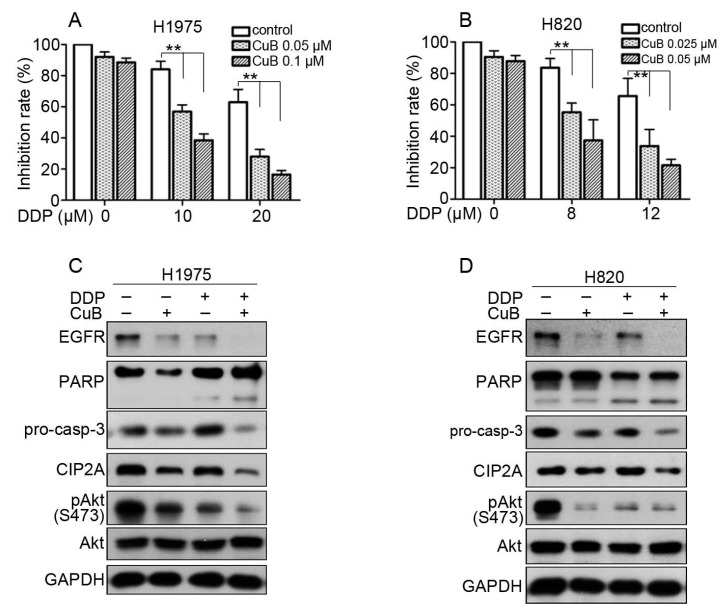
CuB and DDP synergistically inhibit GR NSCLC cells. (**A**,**B**): H1975 and H820 cells were treated for 24 h with DDP in the presence of CuB. MTT assay was used to test the proliferation of cells. * *P* < 0.05, ** *P* < 0.001. (**C**): H1975 cells were cultured with control media, CuB (0.1 μM), DDP (10 μM), or CuB (0.1 μM) plus DDP (10 μM) for 24 h. Cells were then lysed and subjected to Western blot using indicated antibodies. (**D**): H820 cells were cultured with control media, CuB (0.05 μM), DDP (8 μM), or CuB (0.05 μM) plus DDP (8 μM) for 24 h. Cells were then lysed and subjected to Western blot using indicated antibodies.

It has been reported that the combination of chemotherapy and natural compounds can exhibit a synergistic effect to decrease breast cancer cell viability [30]. To explore the inhibitory capacity of a combination of CuB and cisplatin (DDP), we examined cell viability after combined CuB and DDP treatment. CuB plus DDP showed synergistic effects against H1975 and H820 cells (Figure 6A,B). The results from the analysis using CalcuSyn software (version 2.1) showed that the CI value was less than 1 (Table 2). These data suggested that CuB and DDP synergistically suppressed the viability of GR NSCLC cells. DDP further enhanced the CuB-dependent induction of cell apoptosis and its inhibitory effects on EGFR and the CIP2A/Akt pathway (Figure 6C,D). 

### 3.6. CuB Inhibits Tumor Growth In Vivo

**Figure 7:**
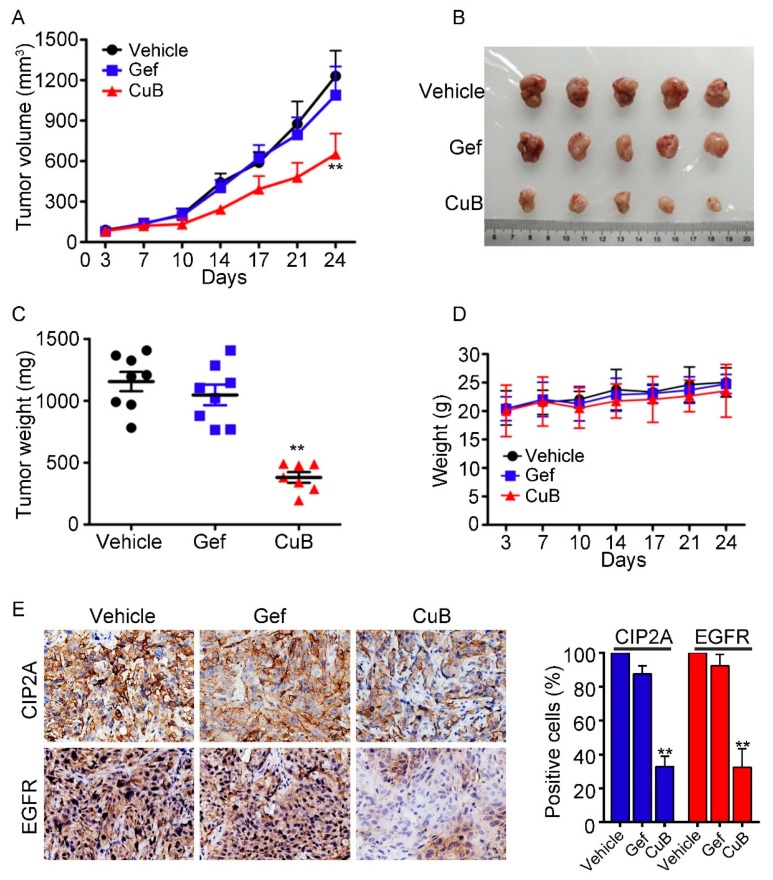
CuB inhibits tumor growth in murine models. (**A**) Murine models were treated with vehicle, gefitinib (Gef, 30 mg/kg), or CuB (0.5 mg/kg) and the tumor volumes were calculated twice a week. ** *P* < 0.01 vs. vehicle or Gef. (**B**) Images of xenograft tumors obtained from mice with different treatment after 24 days. (**C**) Weight of the tumor from each group taken out from the sacrificed mice at the end of the study ** *P* < 0.01 vs. vehicle or Gef group. (**D**) CuB treatment did not affect the murine model body weight. (**E**) The expressions of EGFR and CIP2A in xenograft tumors were analyzed by immunohistochemistry (original magnification 400×), and their expression levels were quantified in percentages of positive cells within five medium-power fields under microscope and shown in histograms; * *P* < 0.05, ** *P* < 0.01 compared with the vehicle group.

To test the in vivo anti-tumor effect of CuB on NSCLC, we implanted 5 × 10^6^ H1975 cells that had been resuspended in 100 μL of RPMI 1640 medium on the right side of nude mice to construct a xenograft mouse model. Treatment began once the tumors reached a palpable size (0.5 cm in diameter). Each of the three groups was administered the vehicle control, gefitinib (30 mg/kg), or CuB (0.5 mg/kg) five times per week for 24 days. The tumor-bearing mice were sacrificed when the tumor reached 1.5 cm in diameter or severe pain diminished their quality of life. We showed that CuB significantly suppressed tumor growth compared to the vehicle control or gefitinib (*P* < 0.01; Figure 7A,B). CuB treatment also markedly decreased tumor weight in the mice (Figure 7C). Importantly, CuB treatment did not significantly decrease the body weight of the mice, suggesting that CuB did not cause evident side effects (Figure 7D). When all of the mice were sacrificed, the tumor specimens were isolated and examined using immunohistochemistry, and the data suggested that the expression levels of CIP2A and EGFR were downregulated in the CuB-treated groups (Figure 7E). Therefore, CuB is predicted to be a potential therapy for GR NSCLC.

## 4. Discussion

CuB has anti-proliferative effects on various lung cancer cell lines in vitro and in vivo [31,32,33]. However, the cytotoxic effects of CuB on EGFR-mutant GR NSCLC cells remain poorly understood. This study reports, for the first time, that CuB suppressed the proliferation of GR NSCLC cells in vitro and in vivo by inducing lysosome-mediated EGFR degradation and by downregulating the CIP2A/PP2A/Akt signaling axis. These results strongly suggest the possible therapeutic value of CuB in patients with GR NSCLC that carry EGFR mutations.

More than 90% of solid tumor deaths are due to tumor metastasis [34]. Thus, inhibiting or preventing cancer metastasis is an important means to improve the survival rate of cancer patients. Our results indicated that CuB significantly suppressed the invasion (Figure 2A) and migration (Figure 2B) of H1975 cells. Escape from apoptosis is an important feature of cancer progression and drug resistance, and the activation of apoptosis has become another important strategy for cancer treatment [35]. Casp-3 is an effector of extrinsic and intrinsic apoptotic signaling [10]. We showed that CuB induced a reduction in the levels of pro-casp-8 and pro-casp-3 and induced the proteolysis of PARP (Figure 2D), which suggested that casp-8 and casp-3 were activated. Thus, CuB may promote apoptosis by activating extrinsic apoptosis signaling, indicated by the activation of casp-8.

The abnormal expression or activation of EGFR and its downstream signaling pathways can promote malignant processes, invasion, and drug tolerance in many human cancers [36]. It is well-known that GR NSCLC cells are largely dependent on the constitutive activity of EGFR kinase signaling. Activation of EGFR, in turn, activates its downstream kinases, such as Akt and ERK, thereby promoting the proliferation and invasion of cancer cells [27]. Given the cytotoxic effects of CuB on GR NSCLC cells, we investigated whether CuB affects EGFR. The results showed that with increased CuB dose and exposure time, the level of EGFR protein was significantly decreased, and the mRNA expression level was not affected (Figure 3A–E). These results suggested that CuB may affect the protein stability of EGFR. A key mechanism for downregulating EGFR signaling is lysosomal-mediated trafficking and degradation [36]. Next, we showed a decrease in EGFR–LAMP-1 colocalization in the absence of CuB (Figure 3F). To further assess whether the CuB-induced degradation of EGFR was mediated by lysosomes, the effects of the lysosome inhibitor chloroquine on CuB-induced EGFR degradation were examined (Figure 3G). The results demonstrated that CuB induced the lysosomal-mediated degradation of EGFR. We next assessed which downstream signaling pathways may have mediated EGFR signaling. ERK is an important downstream signaling protein of EGFR and is involved in the regulation of biological processes such as cell proliferation, invasion, and apoptosis [37]. We found that CuB inhibited ERK phosphorylation (Figure 3H). Therefore, CuB is a novel anti-tumor drug that treats GR NSCLC by inhibiting the EGFR/ERK pathway.

Previous studies have shown that high CIP2A expression was highly correlated with cancer invasiveness and poor prognosis in lung cancer; thus CIP2A is used as a potential molecular marker and therapeutic target for the treatment of lung cancer [38]. We found that CuB was also able to induce a marked dose- and time-dependent reduction of CIP2A at the protein levels in GR NSCLC (Figure 4A,B). Recent studies have implicated CIP2A-mediated increases in Akt activity in the inactivation of PP2A phosphatase activity. Some natural compounds that target the CIP2A protein have shown potential effects on a variety of tumors in vivo and in vitro [39,40]. We next examined the PP2A activity and pAkt levels in GR NSCLC cells after CuB was administered. The data suggested that CuB reactivated PP2A activity and inactivated Akt (Figure 4C–F), indicating that the CIP2A/PP2A/Akt pathway may serve as an alternative mechanism that underlies the effects of CuB.

When CuB was combined with the conventional drug DDP, they had a synergistic cytotoxic effect on GR NSCLC cells (Figure 6). In the H1975 xenograft mouse model, CuB significantly inhibited tumor growth and had little effect on body weight of the mice (Figure 7A–D). Tumor tissues isolated from mice also showed that CuB inhibited EGFR and CIP2A expression in vivo (Figure 7E). Our data suggest that CuB not only directly affects EGFR degradation, but also affects the CIP2A/PP2A signaling pathway. Furthermore, the growth and invasion of GR NSCLC cells were inhibited by CuB activity via the CIP2A/PP2A signaling pathway. Therefore, CuB may become a novel anti-tumor drug for the prevention and treatment of GR NSCLC.

## 5. Conclusions

In conclusion, we reported that CuB significantly suppressed tumor growth and invasion and activated apoptosis in GR NSCLC in vitro and in vivo. Our data further revealed that CuB inhibited ERK and Akt phosphorylation by inducing the lysosomal degradation of EGFR and that CuB inhibits the CIP2A/PP2A signaling axis. These observations indicate that CuB could be a promising therapeutic agent for treating GR NSCLC, and additional toxicological experiments are necessary to verify this conclusion.

## Figures and Tables

**Table 1:** IC_50_ of CuB on GR NSCLC cell lines ^a^.

Cell Lines	16-HBE	H1299	A549	H1975	H820
IC_50_ (μM)	4.23 ± 0.81	0.77 ± 0.04	0.76 ± 0.06	0.63 ± 0.06	0.19 ± 0.04

^a^ The cells were treated with CuB at various concentrations for 24 h, the cell cytotoxicity was analyzed by MTT assay, and the IC_50_ was calculated using CalcuSyn. Values shown are means plus or minus SD of quadruplicate determinations.

**Table 2:** CuB and DDP combination index (CI) values ^a^.

H1975				H820			
CuB (μM)	DDP (μM)	Effect	CI (CuB+DDP)	CuB (μM)	DDP (μM)	Effect	CI (CuB+DDP)
0.05	10	0.43	0.44	0.025	8	0.44	0.51
0.05	20	0.72	0.29	0.025	12	0.66	0.53
0.1	10	0.61	0.28	0.05	8	0.63	0.36
0.1	20	0.84	0.18	0.05	12	0.79	0.39

^a^ H1975 or H820 cells were treated with CuB and DDP combinedly or alone with indicated concentrations for 24 h, the cytotoxicity was analyzed by MTT assay, and the CI values were calculated using CalcuSyn software.
